# Bone and Tooth Regeneration in Maxillofacial Region

**DOI:** 10.1155/2015/934525

**Published:** 2015-09-28

**Authors:** Kazuhisa Bessho, Joo L. Ong, Norbert R. Kübler, John G. Clement

**Affiliations:** ^1^Department of Oral and Maxillofacial Surgery, Graduate School of Medicine, Kyoto University, Kyoto 606-8507, Japan; ^2^USAA Foundation, College of Engineering, The University of Texas at San Antonio, San Antonio, TX 78249, USA; ^3^Department of Oral and Maxillofacial Surgery, Center of Operative Medicine II, Heinrich Heine University, 40225 Düsseldorf, Germany; ^4^Forensic Odontology, Melbourne Dental School, The University of Melbourne, Carlton, VIC 3053, Australia

Autogenous graft is the preferred material of choice due to its excellent clinical outcome and thus has been the gold standard for hard tissue reconstruction, especially for small defects. However, there are drawbacks to the use of autogenous graft materials and these drawbacks include the need for a second surgical site to harvest healthy donor tissues, the limited amount of donor tissues that can be harvested for large defects, and the increased possibility for tissue morbidity, infection, and pain at the second surgical site. Although it is more plentiful to obtain allografts, there is a risk of disease transmission from donor sites when using allogenic grafts for treatment in the clinics. With the advent in biomaterials research, various other sources of graft materials have shown promise for clinical use. As such, the ability of clinicians to evaluate, to understand, and to use other sources of graft materials is a key for successful reconstructions as well as tissue regenerations. This special issue represents a small cross section of some of the graft materials that are currently being investigated for clinical applications. Ranging from synthetic biomaterials to the use of stem cells, these papers also represent the diverse and multidisciplinary approaches that can be adapted for both maxillofacial and orthopedics reconstructions as well as tissue regenerations.

*Kazuhisa Bessho* *Joo L. Ong* *Norbert R. Kübler* *John G. Clement*

